# Treating Imatinib-Resistant Leukemia: The Next Generation Targeted Therapies

**DOI:** 10.1100/tsw.2006.184

**Published:** 2006-08-11

**Authors:** Michael R. Burgess, Charles L. Sawyers

**Affiliations:** ^1^ Molecular Biology Institute, University of California, Los Angeles, CA, USA; ^2^ Howard Hughes Medical Institute, University of California, Los Angeles, CA, USA; ^3^ Division of Hematology and Oncology, Department of Medicine, David Geffen School of Medicine, University of California, Los Angeles, CA, USA

**Keywords:** BCR-ABL, resistance, imatinib, kinase inhibitors, chronic myelogenous leukemia (CML), gastrointestinal stromal tumor (GIST), hypereosinophilic syndrome (HES)

## Abstract

Imatinib (Gleevec/STI-571/CGP57148B, Novartis) is a small-molecule, tyrosine kinase inhibitor developed to target BCR-ABL, c-Kit, and PDGF-R. Through inhibition of these oncogenic kinases, imatinib is effective in the treatment of BCR–ABL—positive leukemia, gastrointestinal stromal tumor, and hypereosinophilic syndrome, respectively. However, clinical success of imatinib is hampered by acquired resistance that may occur through several mechanisms including kinase domain mutation, target amplification, and activation of alternate signaling pathways. Strategies to overcome resistance have included targeting BCR-ABL stability and downstream signaling pathways important for tumor growth. Additional work has shown that new BCR-ABL kinase inhibitors with increased potency or alternate conformation-binding properties can target imatinib resistance. This review focuses on the mechanisms of imatinib resistance and the strategies currently being developed to overcome clinical resistance.

## THE DEVELOPMENT OF IMATINIB

Chronic myelogenous leukemia (CML) is a disorder that results from the malignant transformation of pluripotent hematopoietic stem cells[1]. Clinically, the disease progresses through distinct stages, starting with a chronic phase that progresses into an accelerated, rapidly fatal stage termed “blast crisis”. The disease is characterized by the Philadelphia (Ph) chromosome, a t(9;22) translocation resulting in the expression of BCR-ABL, a constitutively active, cytoplasmic tyrosine kinase[2,3,4]. The t(9;22) rearrangement is present in more than 95% of CML patients and about half of patients with adult-onset acute lymphoblastic leukemia (ALL)[1,5,6].

Multiple experimental models have demonstrated the central role of BCR-ABL in leukemogenesis. *In vitro* culture systems demonstrated that BCR-ABL can transform immature hematopoietic cells, some fibroblast cell lines, and hematopoietic cell lines rendering them growth factor independent[7,8,9,10]. Mouse models demonstrated that BCR-ABL expression in immature hematopoietic cells was sufficient to induce a CML-like syndrome in mice[11,12]. Other mouse models, including transgenic studies, were able to recapitulate at least some of the features of blast crisis[13,14,15,16]. These studies indicated that

BCR-ABL expression in hematopoietic precursors was necessary and sufficient for the induction of leukemia.

Currently, the only known cure for CML is allogenic stem cell transplantation in the chronic phase of the disease. This therapy has demonstrated survival rates of 50–70% after 3–10 years of follow-up[17,18,19]. Unfortunately, this treatment is not an option for the majority of patients due to the lack of an HLA-matched related donor or the risk of transplantation-related mortality. Until recently, the standard treatment for patients unable to undergo allogenic stem cell transplantation was interferon-alpha (IFN-α) alone or in combination with cytarabine[20,21]. While this therapy results in increased survival and a low rate of complete remission, it is associated with a range of adverse effects[22,23,24,25]. Due to the central role of BCR-ABL signaling in CML, investigators questioned whether direct pharmacologic inhibition of BCR-ABL could control the disease. In support of this approach, multiple mouse models of cancer, including a BCR-ABL–driven leukemia model, demonstrated that the initial oncogene, despite the presence of additional genetic hits, was required to maintain the tumor phenotype[16,26,27].

The 2-phenylamino pyrimidine inhibitor imatinib (CGP57148B, STI-571, Gleevec), developed by Novartis, was shown to inhibit a select group of kinases including c-Abl, c-Kit, Arg, and platelet-derived growth factor receptor (PDGF-R)[28,29,30]. Cocrystal studies of the Abl kinase domain with imatinib demonstrated that the inhibitor's specificity could be attributed to binding only the inactive form of the kinase by an induced-fit mechanism[31]. In preclinical models, imatinib inhibited cellular proliferation in BCR-ABL–expressing cells and tumor formation in animal models of CML[32]. In a phase I clinical trial, imatinib was shown to be well tolerated and have substantial activity against Ph-positive CML and ALL[6,33]. However, the clinical success of imatinib was limited early on, especially in blast crisis patients, by the development of resistance during the course of treatment[6]. Complete hematologic and cytogenetic responses were common and durable in chronic phase, but in blast crisis, where only a third of patients demonstrated sustained hematologic responses, the median duration of response was 10 months[34,35,36]. Despite this problem of drug resistance, imatinib has become front-line treatment for Ph-positive leukemia patients who are not candidates for allogenic stem cell transplantation[23,37].

In addition to Ph-positive leukemia, imatinib was shown to be effective in treating gastrointestinal stromal tumor (GIST) and hypereosinophilic syndrome (HES) caused by activated forms of c-Kit and PDGF-R, respectively[38,39]. In the case of GIST, about half of the patients in a clinical trial achieved either a partial or complete response while on imatinib therapy[38]. However, acquired resistance to imatinib is well established in the literature for both of these malignancies[40,41].

## CLINICAL IMATINIB RESISTANCE IS PRIMARILY MEDIATED BY KINASE DOMAIN MUTATIONS

Soon after the clinical introduction of imatinib, resistance to drug therapy was observed in patients due to kinase domain mutation preventing drug binding, or less frequently, by BCR-ABL genomic amplification[42]. The first reported clinical resistance BCR-ABL mutation at T315I (Abl type Ia sequencing numbering) was postulated to block imatinib from binding due to the loss of a drug-kinase hydrogen bond and a steric clash between imatinib and the bulky hydrocarbon isoleucine side chain in the mutant, meanwhile preserving ATP-binding and kinase activity[42]. Multiple subsequent studies have shown that kinase domain mutation is a major resistance mechanism in CML[37,43,44,45,46,47, 48,49,50,51]. In a recent study of 84 Ph-positive leukemia patients resistant or intolerant to imatinib, 71% were shown to have BCR-ABL mutations[52]. Additional work demonstrated analogous resistance mutations in c-Kit and PDGF-R in the course of imatinib treatment of other malignancies[40,41]. Several of the CML studies demonstrated the presence of kinase domain mutations in patients never treated with imatinib, possibly indicating that at least some of these mutations confer increased biological fitness over wild-type BCR-ABL[47,53,54,55]. Mutations arising by chance that increase kinase fitness may allow for clonal expansion of one BCR-ABL mutant even in the absence of drug selection. The reported prevalence of resistance-conferring mutations in CML patients has ranged from 45–90% and represents the most significant mechanism of clinical resistance to therapy[37,44,52].

Following the report of the T315I mutation, other BCR-ABL mutations were uncovered in patients displaying varying degrees of drug resistance[42]. Comparison of the location of these resistance mutations to the cocrystal structure indicated that the majority of resistance mutations in clinical samples did not occur at drug contact residues[31]. Mutations at contact residues were observed including T315I, F317L, and F359V[44]. The remainder of the mutations occurred in the phosphate-binding loop (P-loop) including M244V, G250E, Q252H/R, Y253F/H, and E255K, near the activation loop, including M351T and E355G, and elsewhere in the molecule[44]. Those mutations that did not directly impair drug binding were postulated to prevent conformational changes in the P-loop and activation loop required for imatinib binding. Since imatinib only binds the inactive conformation of the kinase, any mutation that shifts the kinase to the active conformation or impinges on BCR-ABL autoinhibition will limit imatinib from binding[31,44].

This principle, first observed in clinical samples, was further supported by an *in vitro* mutagenesis study of BCR-ABL[56]. In a study by Azam et al., an unbiased genetic screen was performed to identify mutations that confer resistance to imatinib[56,57]. The screen recovered 112 amino acid substitutions at 90 residues[56]. Resistance mutations were recovered throughout BCR-ABL and, consistent with clinical observations, only a minority mapped to drug-kinase contact residues. This observation implies that inhibitors that bind alternate conformations of the kinase may have activity against many imatinib-resistant BCR-ABL mutants.

Acquired resistance to small-molecule, inhibitor therapy mediated by kinase domain mutation is an emerging theme in targeted cancer therapy. Since the original T315I BCR-ABL mutation was described in CML, homologous mutations have been observed in other targeted kinases that mediate resistance[42]. Work from multiple labs has demonstrated acquired-resistance mutations in epidermal growth factor receptor (EGFR) in patients with non-small-cell lung cancer (NSCLC) treated with the EGFR inhibitors gefitinib (Iressa, AstraZeneca) and erlotinib (Tarceva, Genentech)[58,59]. The T790M EGFR mutation reported in NSCLC, conferring resistance to these compounds, is directly analogous to several acquired imatinib-resistant mutations in other kinases: the T674I PDGFR-alpha mutation in HES, the T670I KIT mutation in GIST, and the T315I BCR-ABL mutation first described in CML[40,41,42,59]. Additionally, one study indicated that the T790M EGFR mutation was present in a cell line that had never been treated with any of the EGFR inhibitors[59]. This further raises the possibility that some kinase domain mutations, in addition to mediating resistance, generate kinases of increased fitness.

## PROPOSED MECHANISMS OF RESISTANCE IN THE SETTING OF NONMUTANT BCR-ABL

While BCR-ABL mutation and amplification likely contribute to nearly 90% of clinical imatinib resistance, alternate mechanisms of resistance have been proposed by investigators. One aim has been to identify mechanisms that prevent imatinib from reaching the target. Drug efflux, for example, has often been implicated in resistance to chemotherapeutics. In one study, investigators reported increases in the multidrug resistance (MDR1) gene that encodes the P-glycoprotein (Pgp) in a subclone of a resistant human leukemia line derived by growing the cells in increasing concentrations of imatinib[60]. Increased expression of Pgp could potentially deplete intracellular levels of imatinib, thereby reactivating BCR-ABL signaling. While some *in vitro* models have supported this finding, others have demonstrated that overexpression of Pgp in K562, a human CML cell line, does not confer resistance to imatinib[61,62,63]. These results may indicate that cell lines selected over time for imatinib resistance *in vitro* may not recapitulate actual mechanisms of resistance in patients. Other studies showed that imatinib may be sequestered in the plasma by drug-binding proteins. Plasma protein alpha1 acid glycoprotein (AGP) bound imatinib at physiologic concentrations *in vitro* and blocked the ability of imatinib to inhibit BCR-

ABL kinase activity[64]. Whether drug efflux or plasma sequestration represent major mechanisms of resistance in CML still remains controversial.

Imatinib resistance might additionally result from loss of target dependence through the activation of alternate signaling pathways[65,66]. This possibility is supported by precedent that Src family kinases may operate downstream of BCR-ABL in CML[40,41,67,68]. This BCR-ABL–independent model of resistance relies on the activation of other tyrosine kinases to drive the disease. In an *in vitro* model of resistance, one group demonstrated that an imatinib-resistant K562 subline was sensitive to inhibition of the Src-family kinases Lyn and Hck, and that these kinases were up-regulated in patients who acquired resistance during the course of imatinib therapy[65]. The same group extended these observations to continuous cell lines derived from imatinib-resistant patients[66], but there is little current evidence that Src activation is responsible for true clinical resistance to imatinib in patients. If so, however, such patients may benefit from the current, dual SRC/ABL inhibitors in clinical development[52,69,70]. Future work will demonstrate the significance of BCR-ABL–independent pathway activation and whether inhibition of these pathways is a viable means of targeting imatinib-resistant CML. The proposed mechanisms of resistance to imatinib are summarized in Fig. 1.

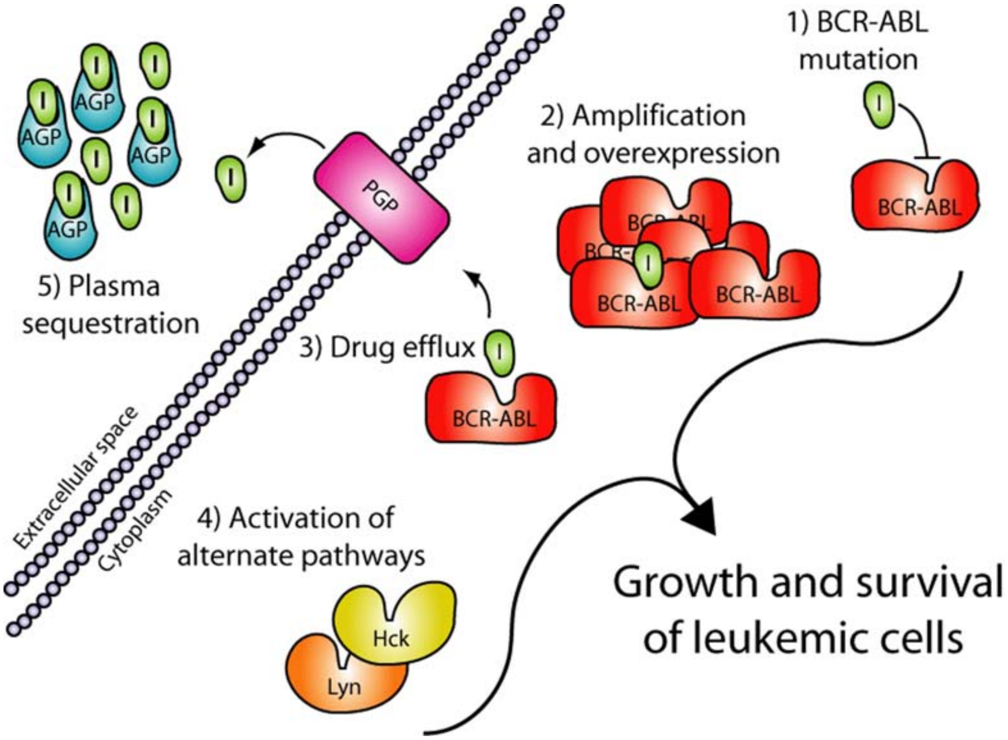

**FIGURE 1.** Mechanisms of imatinib resistance in CML. Clinical imatinib resistance is mediated primarily by (1) kinase domain mutation preventing imatinib from binding. (2) Genomic amplification and protein over expression of BCR-ABL has also been demonstrated in patients resistant to imatinib. Other mechanisms may include (3) drug efflux mediated by Pgp expression, (4) activation of alternate signaling pathways including the Src-family kinases Lyn and Hck, and (5) plasma sequestration by imatinib-binding proteins such as AGP. All mechanisms include the final common pathway of tyrosine kinase activity, promoting growth and survival of the leukemic cells. Imatinib is depicted as a green oval (I).

## STRATEGIES FOR TARGETING IMATINIB-RESISTANT LEUKEMIA

Clinical resistance to imatinib caused by mutation and reactivation of BCR-ABL signaling indicates that alternate therapies that continue to target the BCR-ABL protein should be effective. Multiple studies demonstrate that inhibition of BCR-ABL signaling by targeting protein stability, downstream signaling pathways, or by blocking kinase activity with structurally distinct inhibitors could be effective in imatinib resistance.

Targeting BCR-ABL stability should be an effective therapy when overexpression or mutation of BCR-ABL mediates resistance. BCR-ABL was shown to be a client of the molecular chaperone heat shock protein 90 (Hsp90)[71]. Hsp90 inhibition pharmacologically depleted cells of BCR-ABL protein and increased apoptosis. One study showed that inhibition of Hsp90 with geldanamycin and 17-allylaminogeldanamycin (17-AAG, National Cancer Institute) could induce degradation of imatinib-resistant, mutant BCR-ABL (T315I and E255K)[72]. Encouragingly, despite Hsp90 expression in normal tissue and in tumors, Hsp90 inhibitors may selectively kill cancer cells compared to normal cells because they have higher affinity for the activated conformation of Hsp90 found in malignant cells and therefore may be effective in patients[73]. Formulation and drug delivery issues have hampered the clinical evaluation of these drugs in CML.

Additional studies indicate that inhibition of histone deacetylases (HDAC) may block growth of leukemic cells by multiple mechanisms including decreasing BCR-ABL mRNA and protein levels. The HDAC inhibitor NVP-LAQ824 (Novartis) was shown to have activity against CML blast crisis cells[74]. Additional work demonstrated that HDAC inhibitors given in combination with imatinib may enhance the kinase inhibitor's cytotoxic effects[75,76]. Finally, recent studies using a combination of Hsp90 and HDAC inhibition have shown synergistic activity against both imatinib-sensitive and -resistant human leukemic cell lines driven by BCR-ABL[77].

Since BCR-ABL signaling is reactivated in imatinib resistance, targeting downstream pathways alone or in conjunction with BCR-ABL inhibition might control resistant disease. The Ras/MAPK pathway has been shown to be a critical effector pathway activated downstream of BCR-ABL[78,79,80]. Ras is a prototype, small, GTP-binding protein that when activated by receptor tyrosine kinases in a normal cellular context promotes cell cycle progression[81]. Genetic studies demonstrated that inactivation of Ras blocks BCR-ABL–induced transformation of fibroblasts and hematopoietic cells[82]. Farnesyl protein transferase inhibitors (FTIs) were originally designed to block mutant Ras signaling. However, FTIs also inhibit the growth of transformed cells with wild-type Ras, possibly due to effects on other farnesylated proteins. The FTI lonafarnib (SCH66336, Schering-Plough) was shown to inhibit proliferation in resistant cells and increase imatinib-driven apoptosis in sensitive cells[83]. In addition, lonafarnib was effective in reducing tumor burden in a p190 BCR-ABL mouse model of ALL[84]. These results indicate that FTIs may be effective in the setting of mutation or amplification of BCR-ABL. Other potential targets downstream of Ras include Raf-1 (BAY-439006, Onyx) and Mek (CI-1040, Pfizer), for which there are currently inhibitors in clinical development[85,86].

Targeting the PI3K/Akt pathway downstream of BCR-ABL is another potential therapeutic strategy. Class Ia PI3K proteins are heterodimers consisting of a catalytic (p110) and regulatory (p85) subunit that convert phosphatidylinositol-4,5-bisphosphate to phosphatidylinositol-3,4,5-trisphosphate ($PIP_3$). Accumulation of $PIP_3$ leads to activation of multiple downstream effectors including Akt and mTOR, promoting proliferation and survival[87]. Global inhibitors of the PI3K pathway, such as LY294002 (LY) and wortmannin, are able to block BCR-ABL–mediated transformation of hematopoietic cells[88]. Based on these studies, investigators have shown that it may be possible to target PI3K directly (with LY or wortmannin), Akt (with OSU-03012 [National Cancer Institute] that blocks Akt through PDK-1 inhibition), or downstream signals of Akt, such as mTOR (CCI-779, National Cancer Institute), to overcome imatinib resistance[89,90,92].

## TARGETING KINASE INHIBITOR RESISTANCE WITH ALTERNATE KINASE INHIBITORS

Work from multiple investigators has shown that it is possible to overcome imatinib resistance by using BCR-ABL inhibitors that target the imatinib-resistant mutant forms of the kinase. A significantly more potent (10- to 50-fold) derivative of imatinib, nilotinib (Novartis), was recently shown to have a favorable safety profile in a Phase I trial of Ph-positive leukemia[93,94]. While nilotinib also binds the inactive form of the kinase, this compound is able to target all known imatinib-resistant mutants except T315I[94]. Mutations in the P-loop including L248R, Y253H, and E255K/V were less sensitive to nilotinib than wild-type BCR-ABL, but these mutants may be controlled in patients due to overall increased potency of nilotinib compared with imatinib[94].

Potent inhibitors of the Src-family tyrosine kinases are also able to inhibit BCR-ABL kinase activity[95]. While imatinib has not traditionally been considered an inhibitor of Src kinases, recent work has demonstrated that imatinib inhibits the Src-family tyrosine kinase Lck (and to a lesser degree Frk and Fyn), but not other Src-family members[96]. Structural studies demonstrated that the dual Src/Abl inhibitors, in contrast with imatinib, could bind BCR-ABL in an active conformation[31,97]. Since many of the imatinib-resistant mutants are postulated to prevent the kinase domain from achieving the closed conformation necessary for binding, inhibitors that target the active form of the kinase may be effective in these cases.

This principle was first demonstrated with the pyrido-pyrimidine class of dual Src/Abl inhibitors. Studies demonstrated that PD180970 and PD166326 (Pfizer) were effective in targeting most imatinib-resistant forms of BCR-ABL except T315I[98,99,100]. Additional work indicated that PD166326 had greater antileukemic activity than imatinib in a mouse model of CML[101]. In the case of BCR-ABL–independent imatinib resistance, inhibition of the Src kinases with these compounds may be of additional therapeutic value[102].

Structurally distinct dual Src/Abl inhibitors have since been described. AP23464 (Ariad Pharmaceuticals Inc.), an ATP analog, was shown to be 200-fold more potent than imatinib and able to target many imatinib-resistant forms of BCR-ABL except T315I[103]. Dasatinib (BMS-354825, Bristol-Myers Squibb), yet another dual Src/Abl inhibitor, was recently shown in a Phase I trial to induce hematologic and cytogenetic responses in patients with Ph-positive leukemia resistant or intolerant to imatinib[52]. Dasatinib was shown to target most of the common imatinib-resistant forms of BCR-ABL except T315I[69,70]. The ability of dasatinib to inhibit almost all imatinib-resistant BCR-ABL mutants is likely due to conformation-tolerant binding. Recent cocrystal studies show that dasatinib recognizes multiple states of BCR-ABL[104,105]. Mutations that relieve BCR-ABL autoinhibition and shift the kinase to the imatinib-resistant, active conformation remain sensitive to dasatinib.

To date, all BCR-ABL kinase inhibitors that have been used clinically are not able to inhibit the T315I mutant. Recently, however, an allosteric BCR-ABL inhibitor was described that targets T315I and several other imatinib-resistant mutants[106]. The non-ATP competitive inhibitor, ON012380 (Onconova Therapeutics Inc.), targeted imatinib-resistant forms of BCR-ABL in cell lines and animal models in the low nanomolar range[106]. Additionally, recent work has shown that the Aurora kinase inhibitor VX-680 (Merck) and the p38 inhibitor BIRB-796 (Boehringer Ingelheim), two ATP-competitive compounds, are potential clinical T315I inhibitors[107]. While these results are encouraging, it remains to be determined whether these compounds will be tolerated in patients or have their own distinct set of resistance mutations, and highlights the need for clinical-grade inhibitors against T315I BCR-ABL.

Similarly, targeting mutant kinases with novel inhibitors is likely to be effective in other imatinib-resistant malignancies. PKC412 (Novartis), an alternative inhibitor of PDGFR-alpha and Kit now in clinical trials for FLT3 driven acute myelogenous leukemia (AML), was shown to target the T674I mutation responsible for some clinical imatinib resistance in HES[108]. Additional work demonstrated that PKC412 could target some c-Kit and PDGFR-alpha mutants driving imatinib-resistant GIST[109,110]. Sunitinib (SU11248, Pfizer), a promiscuous compound that targets c-Kit, may also prove to be clinically useful in imatinib-resistant GIST[111]. Table 1 summarizes some of the compounds potentially useful in targeting imatinib-resistant malignancies.

**TABLE 1**
**Summary of Compounds Utilized to Target Imatinib Resistance**

| Mechanism of Action | Compound | Molecular Target(s) | Refs. |
|---|---|---|---|
| BCR-ABL stability | Geldanamycin/17-AAG | HSP-90 | 72 |
| | NVP-LAQ824 | Histone deacetylases | 74 |
| Downstream signaling | | | |
| Ras/MAPK | Ionafarnib (SCH66336) | Farnesyl protein transferase (Ras and other proteins) | 83,84 |
| | BAY-439006 | Raf-1 | 85 |
| | CI-1040 | Mek | 86 |
| PI3K/Akt/mTOR | LY294002/wortmanin | PI3K | 88 |
| | OSU-03012 | PDK-1 (Akt) | 89 |
| | CCI-779 | mTOR | 92 |
| BCR-ABL kinase activity | | | |
| | Nilotinib (AMN107) | BCR-ABL | 93,94 |
| | PD166326, PD180970 | BCR-ABL, Src kinases | 97,98,99,100,101 |
| | AP23464 | BCR-ABL, Src kinases | 103 |
| | Dasatinib (BMS-354825) | BCR-ABL, Src kinases | 52 |
| | ON012380 | T315I BCR-ABL | 106 |
| | VX-680 | Aurora kinase, T315I BCR-ABL | 107 |
| | BIRB-796 | p38, T315I BCR-ABL | 107 |
| Additional Malignancies | | | |
| Imatinib-resistant HES, GIST | PKC412 | FLT3, PDGFR, VEGFR, PKC, c-Kit | 108,109,110 |
| | Sunitinib (SU11248) | FLT3, PDGFR, VEGFR, c-Kit | 111 |

## COMBINATION THERAPY

Multiple studies have suggested that combination therapy of imatinib with conventional chemotherapeutics, drugs that decrease BCR-ABL stability, or inhibitors of downstream BCR-ABL targets could be more effective than imatinib alone[89,90,92,112,113,114,115,116]. Work from our laboratory has shown that imatinib in combination with other BCR-ABL kinase inhibitors that target distinct conformations of the kinase can limit the spectrum and emergence of resistant BCR-ABL clones in an *in vitro* assay and additively inhibit the growth of wild-type BCR-ABL–expressing cells[69]. In patients, treating BCR-ABL with multiple inhibitors could potentially delay the emergence of resistance and reduce disease burden due to the additive effect on wild-type BCR-ABL. Clinical trials that will assess the safety and efficacy of such a strategy seem warranted. While each of these compounds is likely to have its own distinct spectrum of mutations that confer resistance, a combination of three or more inhibitors all targeting BCR-ABL kinase activity may be of benefit in CML. This concept is supported by recent mathematical modeling of drug resistance in CML that predicts multiple non-cross-resistant drugs are needed to prevent treatment failure[117]. Additionally, due to the additive and possibly synergistic effects on wild-type BCR-ABL, such a cocktail is likely to be effective where genomic amplification of *BCR-ABL* contributes to resistance. Imatinib is likely to remain a front-line CML therapy due to its safety and efficacy, but in the near future, we may see that a cocktail of BCR-ABL inhibitors, perhaps in combination with drugs that target downstream effectors, might delay resistance and more effectively control the disease.

## ACKNOWLEDGMENTS

We thank Dr. Brian Skaggs for helpful discussions and critical reading of this manuscript. M.R.B. is supported by the NIH NIGMS training grant, GM08042, the Medical Scientist Training Program, and the Aesculapians Fund of the David Geffen School of Medicine at UCLA. C.L.S. is supported by grants from the Leukemia and Lymphoma Society, is an investigator of the Howard Hughes Medical Institute, and is a Doris Duke Distinguished Clinical Scientist.
